# Capturing a Flavivirus Pre-Fusion Intermediate

**DOI:** 10.1371/journal.ppat.1000672

**Published:** 2009-11-26

**Authors:** Bärbel Kaufmann, Paul R. Chipman, Heather A. Holdaway, Syd Johnson, Daved H. Fremont, Richard J. Kuhn, Michael S. Diamond, Michael G. Rossmann

**Affiliations:** 1 Department of Biological Sciences, Purdue University, West Lafayette, Indiana, United States of America; 2 MacroGenics Inc., Rockville, Maryland, United States of America; 3 Department of Pathology and Immunology, Washington University School of Medicine, St. Louis, Missouri, United States of America; 4 Department of Biochemistry and Molecular Biophysics, Washington University School of Medicine, St. Louis, Missouri, United States of America; 5 Department of Molecular Microbiology, Washington University School of Medicine, St. Louis, Missouri, United States of America; 6 Department of Medicine, Washington University School of Medicine, St. Louis, Missouri, United States of America; University of North Carolina, United States of America

## Abstract

During cell entry of flaviviruses, low endosomal pH triggers the rearrangement of the viral surface glycoproteins to a fusion-active state that allows the release of the infectious RNA into the cytoplasm. In this work, West Nile virus was complexed with Fab fragments of the neutralizing mAb E16 and was subsequently exposed to low pH, trapping the virions in a pre-fusion intermediate state. The structure of the complex was studied by cryo-electron microscopy and provides the first structural glimpse of a flavivirus fusion intermediate near physiological conditions. A radial expansion of the outer protein layer of the virion was observed compared to the structure at pH 8. The resulting ∼60 Å-wide shell of low density between lipid bilayer and outer protein layer is likely traversed by the stem region of the E glycoprotein. By using antibody fragments, we have captured a structural intermediate of a virus that likely occurs during cell entry. The trapping of structural transition states by antibody fragments will be applicable for other processes in the flavivirus life cycle and delineating other cellular events that involve conformational rearrangements.

## Author Summary

West Nile Virus (WNV) and other related viruses such as dengue virus enter their host cell by a process that involves fusion between the viral membrane and the membrane of cellular vesicles (endosomes) resulting in the release of the viral genome into the cytoplasm of the cell. This fusion event is initiated by low pH in the endosomes. Little is known regarding structural changes within the viral particle that render the viral surface proteins capable of fusion. In the present study, we used antibody fragments as a tool to trap virions in a pre-fusion intermediate state and examined these particles by cryo-electron microscopy. We showed that low pH triggered a radial displacement of the virion's external protein layer. The surface proteins moved away from the viral membrane, a shift made possible by the outward extension of a small alpha-helical region of the surface protein. The process gives the proteins greater sideways freedom for their reorganization into the fusion-active state. Our results provide a first structural glimpse into the low pH-induced conformational rearrangement of the flavivirus particle that occurs prior to fusion of viral and endosomal membranes. Such dissection of the fusion process highlights targets for the development of antiviral strategies.

## Introduction

West Nile Virus (WNV), a member of the *Flaviviridae* family, is closely related to other arthropod-borne and medically relevant viruses, such as dengue, tick-borne, Japanese encephalitis, and yellow fever viruses. Flaviviruses are enveloped viruses that enter host cells by receptor-mediated endocytosis. The outer surface of a flavivirus is formed by an icosahedral scaffold of 90 envelope glycoprotein (E) homodimers [1]. The E glycoprotein of flaviviruses has three domains, DI, DII and DIII, with a flexible ‘hinge’ between DI and DII [2]–[4]. An ∼50 amino acid, partially alpha-helical “stem” region connects the E ectodomain with its C-terminal transmembrane anchor. In the mature virion, the stem region lies essentially flat against the viral membrane [5]. Conformational and oligomeric reorganization of E into a fusion-active state occurs during cell entry upon exposure of the virion to the mildly acidic pH in the early endosomes, allowing release of the RNA genome into the cytoplasm. Based on the pseudo-atomic resolution structure of the mature virus at neutral pH [1] and the crystal structure of the solubilized E ectodomain post-fusion trimer [6],[7], mechanistic proposals have been interpolated between these end states as to the rearrangement processes that lead to exposure of the fusion loop on the distal end of the E ectodomain [1], [7]–[9]. From experiments at alkaline pH, a lipid-binding monomeric intermediate form of E has been suggested to precede trimerization [10]. However, no structural intermediates of the fusion process have been captured to date under physiological conditions.

The E glycoprotein is the principal antigen that elicits neutralizing antibodies against flaviviruses [11]. The monoclonal antibody (mAb) E16 neutralizes WNV primarily at a post-attachment stage, probably by interfering with the pH-induced reorganization of E prior to fusion [12]–[15]. In the present study, cryo-electron microscopy (cryoEM) was used to examine WNV complexes with E16 antigen binding fragments (Fab) after exposure to low pH. The virions were trapped irreversibly as a pre-fusion intermediate with the E glycoprotein/Fab layer expanded radially outwards leaving an ∼60 Å-wide gap between the lipid bilayer and the outer protein shell. These structural data suggest that the low pH-triggered formation of fusion-active E homotrimers on the viral surface is preceded by the outward extension of the E stem region.

## Results/Discussion

**Figure 1:**
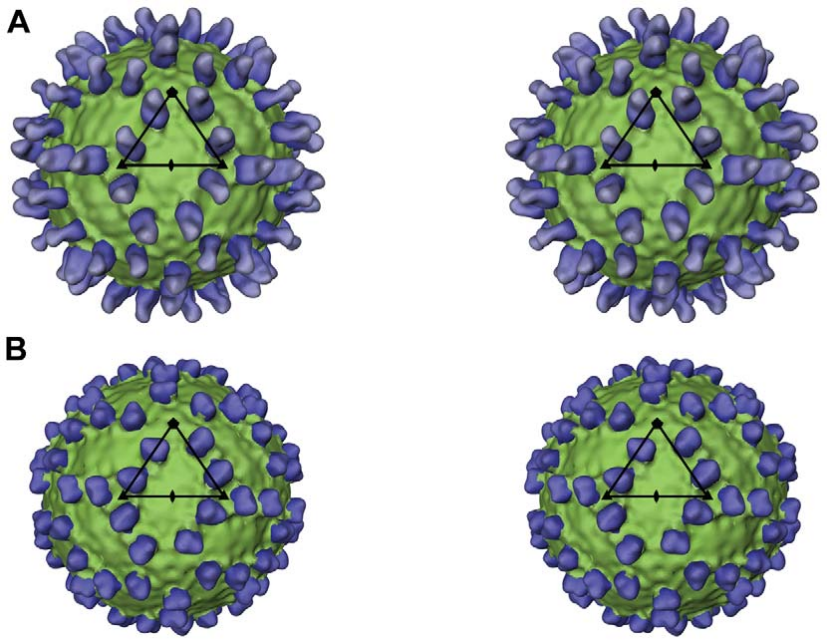
CryoEM image reconstruction of WNV in complex with E16 antibody fragments at pH 8. (A) Stereoscopic view of a surface rendering of WNV (green) complexed with E16 Fab (blue) at 23 Å resolution [14], viewed down an icosahedral twofold axis. The black triangular outline identifies an icosahedral asymmetric unit. (B) Same as A, but depicting the complex of WNV and E16 scFv. E16 scFv binds WNV in a similar fashion as E16 Fab. Only two of three epitopes in the asymmetric unit are utilized. The fivefold proximal epitope is occluded because of steric hindrance.

**Figure 2:**
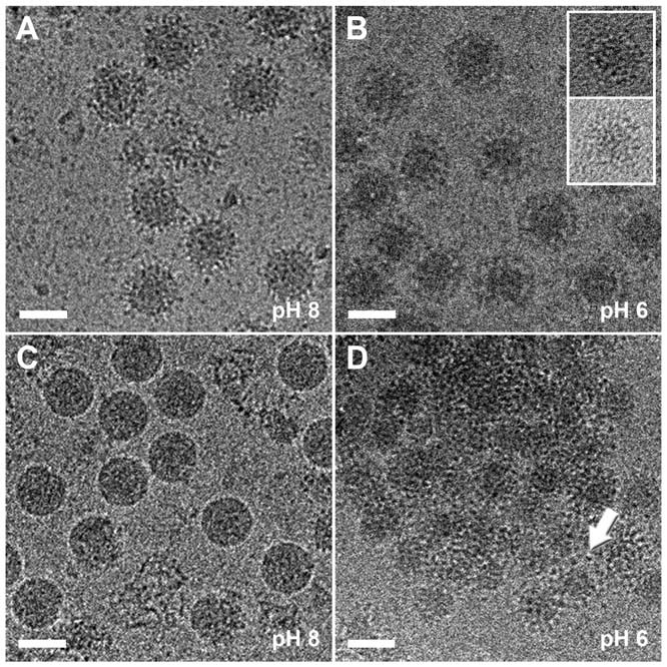
CryoEM images of WNV, alone or in complex with E16 Fab, in different pH environments. (A) WNV/Fab complex at pH 8. (B) WNV/Fab complex at pH 6. Acidification triggered an expansion of the virus particles that resulted in a halo-effect around the dense nucleocapsid core. Inset: Examples of back-neutralized particles showing the irreversibility of the low-pH induced changes. (C) WNV at pH 8. (D) WNV at pH 6. Strong particle aggregation was detected when WNV was exposed to low pH in the absence of E16. Acid-induced structural changes similar to (B) were observed for some virions at the edge of the aggregates, as indicated by the arrow. The scale bars represent 500 Å.

**Figure 3:**
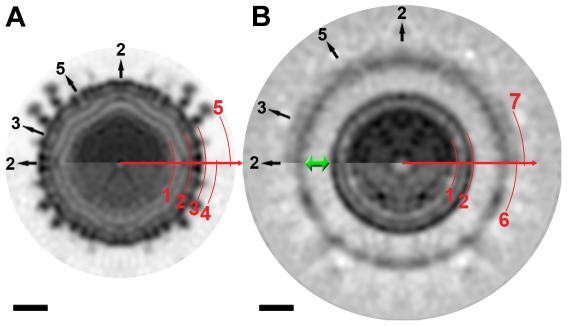
Equatorial slices of cryoEM image reconstructions of WNV in complex with E16 antibody fragments in different pH environments. (A) WNV/Fab complex (top half) and WNV/scFv complex (bottom half) at pH 8, rendered at 23 Å resolution. Red arcs (1 through 5) specify the outer radii of the nucleocapsid core (154 Å), lipid bilayer (205 Å), E glycoprotein shell (247.5 Å), scFv molecules (278 Å), and Fab fragments (318.5 Å) from the viral center. The positions of the icosahedral two-, three- and fivefold axes are indicated with black arrows and numbers. (B) WNV/Fab complex (top half) and WNV/scFv complex (bottom half) at pH 6, rendered to 25 Å resolution. The red arcs (6 and 7) specify the outer radius of the expanded E/scFv (317.5 Å) and E/Fab (347 Å) protein layer, respectively. The low pH triggered radial expansion of the E/scFv or E/Fab protein shell resulted in a ∼60 Å wide shell of low density between the lipid bilayer and the expanded outer protein layer, as indicated by the green arrow. The scale bars represent 100 Å.

Based on crystallographic data and cryoEM reconstructions at neutral pH, we had proposed that E16 neutralizes WNV infection by locking the viral particle in a pre-fusion intermediate state during the structural reorganization of surface proteins that is triggered by endosomal acidification and required for fusion [13],[14]. To evaluate this hypothesis and capture a fusion intermediate, WNV was complexed with E16 Fab (Figure 1A) and shifted from pH 8 to pH 6 *in vitro*, mimicking the low pH environment during cell entry (Figures 2A and 2B). The virions were trapped irreversibly as a pre-fusion intermediate with the E glycoprotein/Fab layer expanded radially outwards by ∼60 Å (Figures 2B and 3). In contrast, the outer radial limits of the nucleocapsid core and lipid bilayer were comparable to those of the mature virus at neutral pH. These results were substantiated when analyzing a complex of WNV with a single-chain antibody derivative (scFv) of E16. At pH 8, the WNV/scFv complex corroborated the E16 binding pattern observed for Fab fragments [14] (Figure 1B). More importantly, exposure to pH 6 resulted in a radial density distribution similar to that of the low-pH structure of the WNV/Fab complex (Figure 3). The expanded outer protein layer of the low pH WNV/scFv complex was found to be thinner than in the Fab complex, consistent with scFv and Fab being external to E and scFv being only about half the size of a Fab fragment. Virion expansion was also observed when WNV was exposed to pH 6 in the absence of bound Fab molecules (Figures 2C and 2D). However, the high degree of aggregation prevented detailed structural analysis. The reduction of particle aggregation at low pH in the presence of Fab is consistent with the hypothesis that E16 blocks the fusion process prior to exposure of the fusion loop [13]–[15].

**Figure 4:**
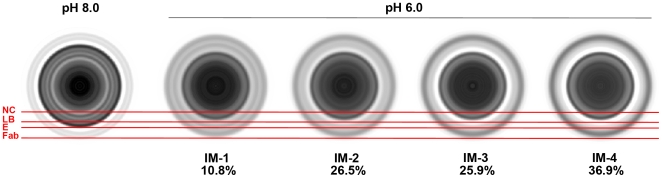
Class averages of radial averaged particle images of the WNV/E16 Fab complex at pH 6.0. The classification suggests the presence of at least four different radial expansion intermediates that represent advancing stages of the E protein layer expansion (IM-1 through 4). The number of images grouped in each class is indicated in percent below the respective class average. Most of the particles clustered in expansion stage IM-4, in which the outer protein layer, presumably composed of E and Fab molecules, reaches its maximum radius separated by an about 60 Å-wide gap from the outer lipid leaflet. A representative class average of the complex at pH 8 is shown on the left. The red lines indicate the radial limits of the nucleocapsid core (NC), the lipid bilayer (LB), the E protein layer (E) and the Fab molecules (Fab) in the pH 8 structure. Areas of high density are depicted with black pixels, low density areas are shown in white.

**Figure 5:**
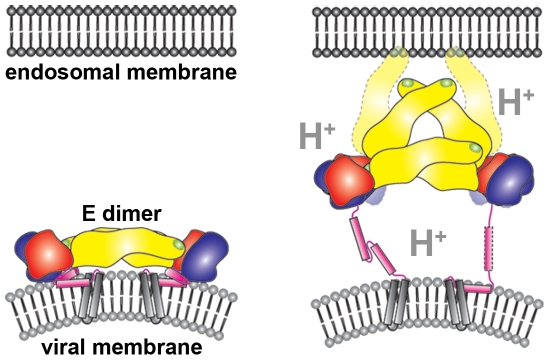
Model of the initial stages of the pH-triggered E rearrangement on the viral surface during flavivirus cell entry. Domains DI, DII, and DIII of E are colored red, yellow, and blue, respectively. The partially alpha-helical stem region is shown in magenta and the transmembrane helices in grey. The fusion loop at the distal end of DII is indicated in green. Low endosomal pH weakens inter- and intramolecular E contacts and induces the outward extension of the whole stem region or its N-terminal portion including helix H1, followed by the dissociation of the E dimers. Further structural repositioning of the E monomers allows the interaction with the endosomal target membrane via the fusion loop. E16 was shown to inhibit the formation of fusion-active E trimers, most probably by interfering with the rearrangement of the E domains as a consequence of adding mass to DIII and sterically clashing with neighboring E and Fab molecules. However, E16 may also sterically prohibit the contact of E with the target membranes.

The density distribution within the outer density shell was not sufficiently resolved to be interpreted in detail. This may be due to heterogeneity of the sample, for example the icosahedral symmetry could be partially lost during the low pH-induced rearrangement and/or the particles might have been captured at different stages of the radial expansion process (Figure 4). Nevertheless, the well-defined ∼60 Å-wide gap between the lipid bilayer and the outer protein layer is presumably traversed by the linearly extended E stem region (Figures 3 and 5). This distance is similar to the ∼58 Å length of the stem helices [4],[5] and the ∼50–60 Å stem length predicted from the post-fusion E trimer structure [6],[7]. The extension of the stem region as being an early event in the membrane fusion process was previously only speculated [7],[16]. This conformational change gives the previously tightly packed E molecules greater lateral freedom for their rearrangement into fusion-active homotrimers.

By using fragments of a neutralizing antibody, we captured a structural intermediate that likely occurs during cell entry when flaviviruses transit through the acidic pH environment of early endosomes. To our knowledge, this is the first structural data on a cell-entry intermediate state of a virion. The low pH-triggered formation of fusion-active E homotrimers on the viral surface is preceded by the outward extension of the stem region (Figure 5). The trapping of structural transition states by antibody fragments may be applicable in the search for other intermediates in the flavivirus life cycle as well as other dynamic processes.

## Materials and Methods

### WNV Propagation and Purification

WNV was propagated in Vero cells using MEM media supplemented with 10% fetal calf serum, non-essential amino acids and glutamine according to standard cell culture procedures. Confluent cells were infected with WNV (New York 1999) at a multiplicity of infection of 0.5 in the presence of 2% fetal calf serum. Cell culture supernatant was harvested 30 h after infection and the virus was concentrated by polyethylene glycol precipitation using PEG 8,000 at a final concentration of 8%. Further purification was achieved by sedimentation through a 20% sucrose cushion, followed by density gradient centrifugation using a 10–30% tartrate step gradient (125,000×g for 2.5 h at 4°C) or by tartrate gradient centrifugation only. The virus fraction was recovered from the gradient, transferred into NTE buffer (12 mM Tris-HCl, pH 8.0, 120 mM NaCl, 1 mM EDTA), and concentrated using Amicon centrifugal filters (Millipore, Billerica, MA, USA). The protein concentration and purity of the final virus preparations were estimated by SDS gel electrophoresis with Coomassie Blue-staining.

### Generation of Mouse E16 Fab and E16 scFv

Fab fragments of the mouse mAb E16 were generated after papain digestion and purified as described [14]. A plasmid encoding the mouse E16 mAb sequence [12] was used as template for the construction of the E16 scFv. Primers H9 (5′ CGAGCTAGCT GAGATCACAG TTCTCTCTAC 3′) and Igh283R (5′ gaaccgccac ctccagaacc gcctccacca ctagcTTTCA GCTCCAGCTT GGTCCCAGC 3′) were used to amplify the recombinant signal sequence and variable domain of the mouse E16 light chain (VL), and primers Igh282F (5′ ggttctggag gtggcggttc tggcggtggt ggatctCAGG TTCAGCTGCA GCAGTCTGG 3′) and Igh284R (5′ tatcaaatgc ggccgcTGAG GAGACTGTGA GAGTGGTGCC 3′) were used to amplify the variable domain of the mouse E16 heavy chain gene sequence (VH). Subsequently, the VL and VH sequences were linked together by overlapping PCR. The product was cloned using NheI and NotI sites into a pCI-neo vector, which contains C-myc and S-tag coding sequences. The mouse E16 scFv contains C-terminal C-myc and S-tags and was expressed in 293H cells. E16 scFv was purified sequentially by DIII (amino acids 296–415 of WNV E protein)-Sepharose affinity and size exclusion chromatography. Briefly, E16 scFv conditioned media was loaded directly onto a DIII-Sepharose column at a flow rate of ∼50 cm/h. The column was equilibrated and washed in phosphate buffered saline, pH 7.2. The protein was eluted in 50 mM glycine, pH 3, and neutralized immediately with 1 M Tris-HCl at pH 8.0. E16 scFv was further purified by size exclusion chromatography (Superdex 75 H/R 10/30, GE Healthcare). The eluate was concentrated using a centrifugation type concentrator (Vivaspin 20, 10 kD molecular weight cutoff) to less than 0.5 ml prior to loading the size exclusion column equilibrated in PBS. Samples were run at a linear velocity of 10 cm/h and the E16 scFv peak was collected, filtered (0.2 mm) and stored at 4°C.

### Complex Formation, CryoEM, and 3D Image Reconstruction

Purified WNV particles were incubated with E16 Fab or E16 scFv in the presence of 100 mM NaCl at 4°C overnight using a ratio of ∼5 Fab or scFv fragments per E molecule. The pH of the sample was lowered to pH 6 (“low pH”) using MES-HAc buffer (0.1 M, pH 4.5) and incubated at room temperature for 5–15 min before flash-freezing. A portion of the low-pH sample was back-neutralized to pH 7.5 using 1 M HEPES buffer, pH 8.0.

Small aliquots of sample were applied to 400 mesh copper grids coated with a holey carbon film and rapidly frozen by plunging into an ethane slush. Micrographs of the frozen-hydrated samples were recorded on Kodak (Rochester, NY) SO-163 films with a CM300 FEG transmission electron microscope (Philips, Eindhoven, The Netherlands) at a total electron dose between 17.2 and 23.4 e^−^/Å^2^.

Image analysis and 3D image reconstructions were performed independently for each dataset. The structure of the WNV/scFv complex at pH 8.0 was determined as described previously for the WNV/Fab complex [14]. A total of 1,139 particles were selected from 53 micrographs (defocus level range 2.74–1.35 µm) and, of those particles, 783 images were used to calculate the final 3D reconstruction with an estimated resolution of 22.8 Å (Figures 1B and 3A). The glycoprotein shell and the two membrane leaflets were clearly resolved, an indication of the good quality of the map. The cryoEM density map was deposited in the EM databank under accession number EMD-5115.

Reconstructions of the low pH complexes, assuming icosahedral symmetry, were computed from several independent datasets (Table 1) as described previously [14] or using a modified version of XMIPP [17]. Starting models for orientation and center search were produced either by assigning random orientations to a subset of images or from best five-, three- and twofold rotational symmetry views using the program *starticos* of the EMAN package [18]. Iterative processing through search and reconstruction cycles was continued on low-pass filtered (15 Å) images until no further improvement of the resulting map was achieved. The resolution of maps, estimated by comparing structure factors for the virus shell computed from two independent half-data sets, ranged from 30 to 50 Å at a Fourier shell correlation coefficient of 0.5. For display, maps were computed using data to a resolution of 25 Å (Figure 3). Of all the low-pH reconstructions, only the maps of the WNV/scFv complex showed the separated leaflets of the lipid bilayer. The lateral density distribution in the expanded outer protein shell of the resulting low-pH structures varied depending on data set, initial model, processing parameters, and reconstruction program used, whereas the radial density distributions were consistent with one another. The lack of consistency of the density distribution in the outer protein layer may be due to heterogeneity of the sample, for example the icosahedral symmetry could be partially lost during the low pH-induced rearrangement and/or the particles might have been captured at different stages of the radial expansion process. To investigate the presence of the latter, radial averages of the particle images were produced and subsequently subjected to a reference-free classification procedure using the program *startnrclasses* of the EMAN package. The classification produced a total of 17 classes for the WNV/Fab complex at pH 6.0, which could be combined into 4 classes (IM-1 through 4) by visual inspection (Figure 4). Of 1,832 particles, 10.8%, 26.5%, 25.9%, and 36.9% were classified into classes IM-1, IM-2, IM-3 and IM-4, respectively. These classes appeared to represent successive stages of a radial particle expansion. Fewer classes were observed for WNV/scFv at pH 6.0, with the majority of images being combined into a class similar to IM-4 of the WNV/Fab complex (data not shown). However, 3D image reconstructions of the individual classes did not lead to a consistent, interpretable density of the outer protein layer, suggesting that the radial expansion is accompanied by the destruction of icosahedral symmetry.

**Table 1:** CryoEM data overview for the WNV/Fab E16 and the WNV/E16 scFv complexes at pH 6.

Specimen	WNV+E16 Fab	WNV+E16 scFv
No. of datasets	3	2
No. of micrographs	27/28/40	49/23
Defocus level range (µm)	3.25–1.61/2.85–1.62/3.14–2.03	3.37–1.88/2.98–2.03
Pixel separation on the specimen^a^	2.97 Å	2.69 Å
No. of particles selected from micrographs	632/658/545	203/177

a The cryoEM micrographs were digitized at 7-µm and 6.35-µm intervals, respectively, using a Zeiss SCAI or Nikon 9000 scanner. Sets of four pixels were subsequently averaged.
